# Anti-Fatigue and Exercise Performance Improvement Effect of *Glossogyne tenuifolia* Extract in Mice

**DOI:** 10.3390/nu14051011

**Published:** 2022-02-27

**Authors:** Yi-Ju Chen, Rathinasamy Baskaran, Marthandam Asokan Shibu, Wan-Teng Lin

**Affiliations:** 1 Department of Surgery, Taichung Veterans General Hospital, Taichung 40704, Taiwan; chenyiju5668@gmail.com; 2 Department of Animal Science and Biotechnology, Tunghai University, Taichung 40704, Taiwan; 3 Department of Bioinformatics and Medical Engineering, Asia University, Taichung 413305, Taiwan; baskaran@asia.edu.tw; 4 Department of Biotechnology, Bharathiar University, Coimbatore 641046, Tamil Nadu, India; shibu.m.a@gmail.com; 5 Department of Hospitality Management, College of Agriculture, Tunghai University, Taichung 40704, Taiwan

**Keywords:** *Glossogyne tenuifolia*, exercise, forelimb grip strength, lactate, ammonia, creatine kinase

## Abstract

*Glossogyne tenuifolia* (GT) is a native perennial plant growing across the coastline areas in Taiwan. The current study aimed to examine the efficacy of GT extract in ameliorating physical fatigue during exercise and increasing exercise performance. Fifty male Institute of Cancer Research (ICR) mice were randomly segregated into five groups (*n* = 10) to GT extract orally for 4 weeks, at different concentrations (50, 100, 250, and 500 mg/kg BW/day): LGT 1X, MGT 2X, HGT 5X, and HGT 10X groups. Forelimb grip strength, endurance swimming time, serum biochemical marker levels, blood lipid profile and histological analysis of various organs were performed to assess the anti-fatigue effect and exercise performance of GT extract. The forelimb-grips strength and endurance-swimming time of GT-administered mice were increased significantly in a dose-dependent manner when compared to the control. Serum glucose, creatine kinase, and lactate levels were increased significantly in the HGT 10X group. Liver marker serum glutamic-oxaloacetic transaminase (GOT) was increased in the HGT 5X and HGT 10X groups, whereas Serum Glutamic Pyruvic Transaminase (GPT) was not altered. Renal markers, creatinine and uric acid levels, were not altered. Muscle and hepatic glycogen levels, which are essential for energy sources during exercise, were also significantly increased in a dose-dependent manner in all GT extract groups. No visible histological aberrations were observed in the vital organs after GT extract administration. The supplementation with GT extract could have beneficial effects on exercise performance and anti-fatigue function without toxicity at a higher dose.

## 1. Introduction

Fatigue is defined as the inability to maintain power output and strength, impairing physical performance. Redundant physical load, insufficient rest, and mental stress/pressure are the causative factors of physiological fatigue, which is further divided into central and peripheral fatigue [1]. Factors affecting the brain, spinal cord, and motor neurons in the central nervous system could result in central fatigue, whereas peripheral fatigue results from muscle weakness induced by changes at the neuromuscular junction [2]. A decline in functional performance could also be associated with physiological fatigue [3]. According to the exhaustion theory, a decrease in energy sources, such as glucose, and accumulation of metabolites due to physical performance are causative factors of fatigue [4].

Fatigue is associated with high-intensity exercise-induced exhaustion, suggesting that the functioning muscle capacity has been substantially impaired [5]. During high-intensity exercise, the body utilizes key energy sources, such as glucose, hepatic, and muscle glycogen; on the other hand, the aggregation of metabolites, such as lactic acid, ammonia, blood urea nitrogen (BUN), and inorganic phosphorus, results in intracellular acidosis, which contributes to muscle fatigue [6]. A decrease in energy sources, such as glucose and liver and muscle glycogen, and accumulation of metabolites, such as lactic acid and ammonia, are the key factors in peripheral fatigue. Peripheral fatigue can be reversed by sustaining the availability of energy sources and clearing the metabolic products [7]. Chronic low-grade inflammation has also been linked to a wide spectrum of chronic illnesses that are characterized by fatigue. Lower stamina and fatigue may occur from an energy deficit caused by mitochondrial dysfunction. Inflammation has been linked to mitochondrial dysfunction and oxidative stress [8]. Recovery from exercise-induced fatigue needs the repair of muscular injury by the metabolites, as well as the removal of metabolites produced during exercise. As a result, frequent exercise coupled with a well-balanced diet can help to prevent muscle fatigue during exercise [9]. In particular, when the intake of dietary protein and energy fail to meet individual demands, body fat and muscle are catabolized to provide energy, leading to symptoms such as fatigue [10]. Previous studies have shown that natural products boost athletic performance and decrease or prevent fatigue without causing adverse effects [11].

**Figure 1:**
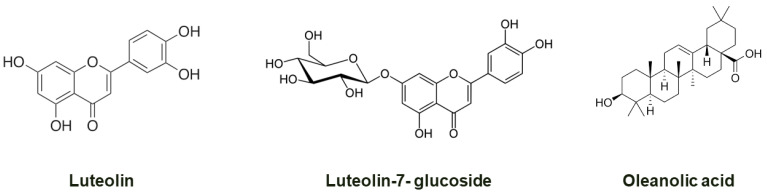
Major compounds present in GT extract. Structure of luteolin, luteolin-7-glucoside, and oleanolic acid.

The medicinal properties of many Chinese herbal teas or medicines are known and include being rich in antioxidants, notably phenolics, alkaloids and antioxidant vitamins [12,13]. The *G. tenuifolia* perennial herb belongs to the Asteraceae family growing in South Asia and Australia. *Glossogyne tenuifolia* (GT) originated from coastal areas of Penghu Islands, Taiwan. In Taiwan, the GT plant was used for preparing herbal tea and in folk medicines. Aqueous extract of dried GT powder is used as an antipyretic, hepatoprotective, and anti-inflammatory medicine in traditional Chinese medicine [13,14,15,16]. Herbal teas/drinks prepared using GT have been used to prevent sunstroke [17]. In our previous studies, we showed that GT extract has antioxidant and hepatoprotective activities against acetaminophen-induced hepatotoxicity and high-fat-diet-induced diabetes [14,18]. Furthermore, we have also shown the hypoglycemic and hypolipidemic effects of GT extracts in animal models [13,19]. We have also shown the presence of major phenolic compounds, such as luteolin, luteolin-7-glucoside, and oleanolic acid, in GT extract by HPLC analysis Figure 1 [14,18,20].

However, the anti-fatigue effect and exercise performance of GT extract were not reported previously. The purpose of the present study was to assess the potential beneficial effects of GT extract on anti-fatigue and ergogenic functions following physiological challenges.

## 2. Materials and Methods

### 2.1. GT Extract Preparation and HPLC Analysis

GT plant was procured from Kaohsiung District Agricultural Improvement Station in Penghu, Taiwan, Republic of China (ROC). The plant was identified by a taxonomist at Tunghai University, Taichung City, Taiwan, ROC, and a specimen of the plant was deposited in the Tunghai University Taichung City, Taiwan, ROC. GT extract was prepared based on our previous study [18]. Safety reports from the Department of Agricultural Pharmaceutical Drug Test, Executive Yuan, Kaohsiung, have shown that Lethal dose 50 (LD_50_) of GT extract is higher than 10 g/kg BW.

### 2.2. Animal Experiments

Male Institute of Cancer Research (ICR) mice were used in the present study. Eight-week-old male ICR mice were procured from BioLASCO, Taiwan. Animals were maintained in the university animal house with a constant temperature of 25 ± 1 °C under the 12:12 h light–dark cycle. Animals were freely allowed to access food and water. Standard commercial laboratory mice feed (PMI Feeds, Inc., Brentwood, MO, USA) and water ad libitum were used. After 1 week of acclimatization, mice were randomly divided into 5 groups (*n* = 10). Control group received vehicle treatment, LGT 1X—low-dose GT extract (50 mg/kg BW/day), MGT 2X—medium-dose GT extract (100 mg/kg BW/day), HGT 5X—high-dose GT extract (250 mg/kg BW/day), and HGT 10X—high-dose GT extract (500 mg/kg BW/day). GT extract was administered for 28 days. All animal treatment procedures were performed in accordance with the Guide for Care and Use of Laboratory Animals (National Institutes of Health Publication NO 85-23, raised 1996). Animal-experiment procedures in the present study were approved by the Institutional Animal Care and Use Committee (IACUC) of Tunghai University.

At the end of treatment, mice were euthanized by 95% CO_2_, blood was collected, and serum was separated by using centrifugation and stored at −80 °C. Organs, such as the liver, lung, heart, kidney, adipose tissue, and skeletal muscles, were collected, weighed, and stored at −80 °C. A small portion of tissue sections was stored in formalin for histological analysis. A small portion of liver and muscle tissue used for estimating glycogen content was washed in saline and stored separately.

### 2.3. Forelimb Grip Strength Test

For estimating the forelimb grip strength of the mice in the different treatment groups, low-force testing system (Model-RX-5, Aikoh Engineering, Nagoya, Japan) was used. Only the front paws of the mice were permitted to grab the pull bar on the grip wire, which was gradually drawn back until they lost their grip on the metal bar. A detailed experiment procedure was described in the previous study [21]. The grip strength of each mouse has measured 10 times, and the longest duration in each trial was recorded.

### 2.4. Weight-Loaded Swimming Test

A weight-loaded swimming test in the mice was performed to evaluate the exercise endurance time based on the previous report [22]. After GT-extract administration, a weight (5% of the mice body weight) was attached to the tail and allowed to swim in a small plastic water tank with a depth of 30 cm of warm water (28 °C). Mice were considered to be exhausted when they failed to rise above the water surface in 10 s.

### 2.5. Biochemical Parameters Associated with Fatigue

After 1 h of GT extract administration, animals were allowed for a swimming test for 15 min without weight. The blood sample was collected after swimming exercise from the submandibular duct of mice, serum was separated by centrifugation and used to analyze fatigue-associated biochemical parameters, such as serum glucose, BUN (blood urea nitrogen), lactic acid, ammonia, CK(creatine kinase) and LDH(lactate dehydrogenase), using an autoanalyzer (Hitachi 7060, Hitachi, Tokyo, Japan).

### 2.6. Serum Marker Analysis

At the end of the experimental period, all mice were euthanized by using 95% CO_2_. Then the blood was collected, and the serum was separated by centrifugation. Biochemical markers, such as GOT, GPT, creatinine, CK, uric acid, LDH, CPK(creatine phosphokinase), total cholesterol (TC), TG (triglycerides), HDL (high-density lipoprotein), LDL (Low-density lipoprotein), BUN, ammonia, and glucose, were measured by using an autoanalyzer (Hitachi 7060, Hitachi, Tokyo, Japan).

### 2.7. Tissue Glycogen Analysis

Liver and skeletal muscles are the two primary sites for glycogen storage; we tested the effect of GT extract administration on the liver and skeletal muscle tissues. Glycogen content in the liver and muscle tissues was quantified based on the previous report [23].

### 2.8. Histological Analysis

Liver, skeletal muscle, heart, lung, kidney, pancreas, and adipose were collected from all treatment groups. A small portion of tissue was fixed in 10% neutral buffered formalin and covered with wax. Then 0.2 µm–size sections were cut from paraffin-embedded tissue blocks, using a microtome. Slides were prepared by deparaffinization, stained with hematoxylin and eosin (H&E), dehydrated through a series of graded alcohols (100%, 95%, and 75%), and rinsed twice in xylene. Photomicrographs were obtained by using a Zeiss Axiophot microscope (Carl Zeiss Microscopy, Thornwood, NY, USA).

### 2.9. Statistical Analysis

Data analyses were performed by using SPSS 17 software (SPSS Inc. Released 2008. SPSS Statistics for Windows, Version 17.0. Chicago, IL, USA). The results shown are the mean ± SEM of three independent experiments. Statistical analysis was performed by one-way analysis of variants. The level of statistical significance was set at *p* < 0.05.

## 3. Results

### 3.1. Effect of GT Extract on Body Weight and Organ Weight

After 28 days of treatment with low, medium, and high dosages (LGT 1X, MGT 2X, HGT 5X, and HGT 10X) of GT extract, the final-day bodyweight of mice was measured. The bodyweight of GT-administered mice was not significantly different when compared to control. Food and water intake were also significantly changed in the GT-treated group and control (Table 1).

### 3.2. GT Extract Increases Forelimb Grip Strength and Endurance-Swimming Time in a Dose-Dependent Manner

**Figure 2:**
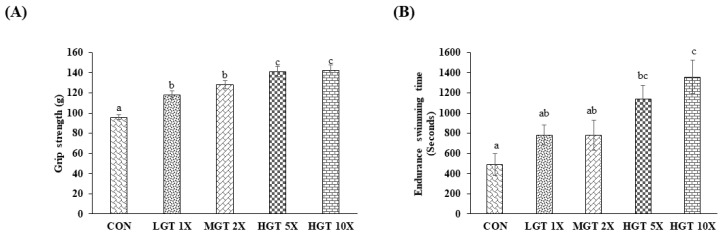
GT extract increases forelimb grip strength and endurance swimming time in a dose-dependent manner: (**A**) forelimb grip strength and (**B**) endurance swimming time. Data are the mean ± SEM for *n* = 10 mice in each group. Values in the same row with different superscript letters (a, b, and c) differ significantly, *p* < 0.05.

The forelimb-grip-strength test in animal models measures the changes in neuromuscular coordination, the intensity of muscle, and total functional capability. The forelimb-grip strength of the control group was around 95.8, which was found to be increased after GT treatment. Grip strength of the animals increased significantly in a dose-dependent manner, where maximum forelimb grip was achieved in the HGT 5X and HGT 10X groups (Figure 2A). Another parameter used for assessing exercise endurance is the duration of the workout/exercise. It is used for testing natural compound ability in alleviating fatigue in high-intensity exercise performance. Endurance-swimming time of the different treatment groups was shown in Figure 2B. Endurance-swimming time of the mice was increased in all GT-extract-administered groups. Low and medium GT doses (LGT 1X and MGT 2X) significantly increased the endurance time. However, high doses of GT (HGT 5X and HGT 10X) showed significantly increased endurance-swimming time than the control and low and medium GT doses.

### 3.3. Effect of GT Extract on Biochemical Markers Associated with Fatigue

**Figure 3:**
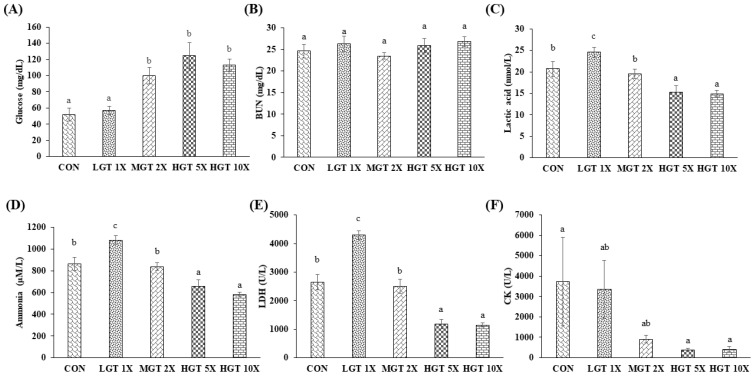
Effect of GT extract on biochemical markers associated with fatigue: (**A**) glucose, (**B**) BUN, (**C**) lactic acid, (**D**) ammonia, (**E**) LDH, and (**F**) CK. Data are the mean ± SEM for *n* = 10 mice in each group. Values in the same row with different superscript letters (a, b, and c) differ significantly, *p* < 0.05.

Figure 3 shows the effect of GT extract treatment on fatigue-associated biochemical parameters, such as serum glucose, BUN, lactate, ammonia, CK, and LDH, after swimming exercise. Levels of serum glucose, BUN, lactic acid, ammonia, CK, and LDH in the blood were quantified to evaluate the anti-fatigue effect of GT extract. Medium and high doses of GT extract significantly increased the glucose level in the blood when compared to control. However, low-dose GT extract has no significant changes in glucose level compared to the control. GT extract has no effect on the BUN level. Low dose and medium doses (LGT 1X and MGT 2X) do not significantly reduce the lactate and ammonia levels, whereas high doses of GT (HGT 5X and HGT 10X) significantly lowered the levels of lactic acid and ammonia after exercise. Creatinine kinase and LDH levels in the blood indicate tissue/muscular damage after exercise. In our present study, HGT 5X and HGT 10X groups significantly lowered the enzyme activity of CK and LDH more than the control and low- and medium-GT doses (LGT 1X and MGT 2X).

### 3.4. Effect of GT Extract on Liver and Muscle Glycogen

**Figure 4:**
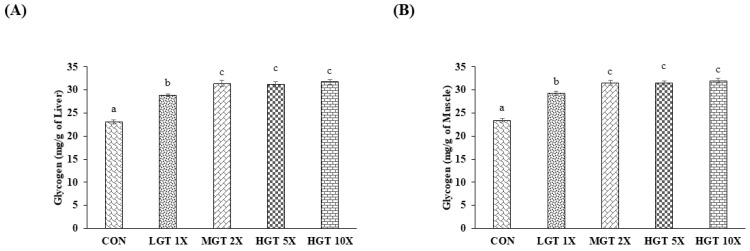
Effect of GT extract on liver and muscle glycogen: (**A**) liver and (**B**) muscle. Data are the mean ± SEM for *n* = 10 mice in each group. Values in the same row with different superscript letters (a, b, and c) differ significantly, *p* < 0.05.

Glycogen is a key energy source utilized during exercise, and higher tissue glycogen levels in the liver and muscle promote physical performance. Estimating the tissue glycogen level helps in identifying the severity of fatigue. GT extract significantly increased the liver and muscle glycogen level. However, MGT 2X, HGT 5X, and HGT 10X groups show significantly higher tissue glycogen levels than the low-dose GT (LGT 1X) group (Figure 4).

### 3.5. Effect of GT Extract on Biochemical Parameters at the End of the Treatment Period

At the end of the treatment period, biochemical parameters for liver, kidney, heart, and muscle injury markers, such as GOT, GPT, creatinine, uric acid, LDH, and CPK; lipid profile; glucose; BUN; and ammonia levels, were quantified to evaluate the anti-fatigue effect GT extract (Table 2). Liver markers GOT and GPT, kidney markers creatinine and uric acid, heart, and muscle injury markers LDH and CPK do not significantly change after GT treatment. In lipid profile total cholesterol, triglyceride and HDL levels were not significantly altered in GT treated group; however, GT extract (LGT 1X, MGT 2X, HGT 5X, and HGT 10X) decreased the LDL level significantly in a dose-dependent manner. No significant changes were observed in glucose, BUN, and ammonia levels in all the groups.

### 3.6. Effect of GT Extract on Histology in Various Organs

**Figure 5:**
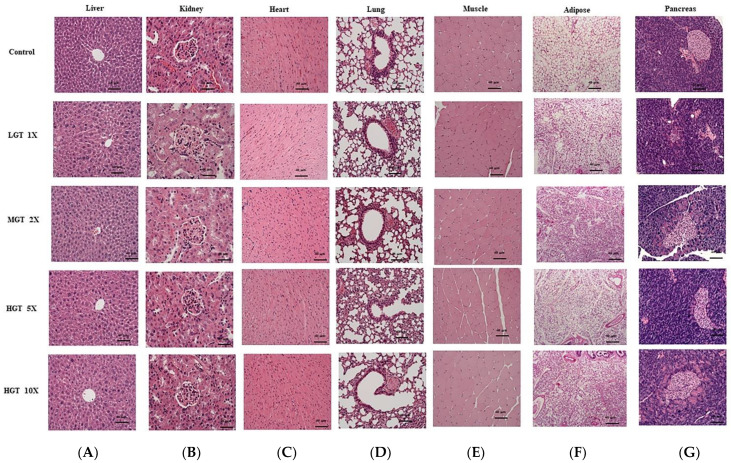
Effect of GT extract on histology in various organs: (**A**) liver, (**B**) kidney, (**C**) heart, (**D**) lung, (**E**) muscle, (**F**) adipose, and (**G**) pancreas. H&E stain (100×).

A histological analysis of various organs, such as the liver, kidney, heart, muscle, lungs, adipose tissue, and pancreas, was performed to understand the morphological damage in the tissues. Even high doses of GT extract (HGT 5X and HGT 10X) do not induce any morphological changes in the liver, kidney, lungs, and muscle. Heart and muscle sections also display normal cellular architecture in GT-treated groups. The morphology of pancreas and adipose tissue do not alter after GT treatment (Figure 5).

## 4. Discussion

Endurance-swimming time, forelimb-grip strength, and changes in biochemical marker levels were studied and a histological analysis of muscle and other tissues was performed to assess the fatigue in animal models [24,25]. Endurance-swimming time in a forced-swimming test, forelimb-grip test, and rotary rod test have been widely used in animal models for evaluating the anti-fatigue efficacy of drugs or natural compounds [26,27]. The enhancement of exercise endurance time is the key manifestation of the anti-fatigue effect of the drug or natural compounds. In our present study, GT-extract administration increased the forelimb-grip strength significantly compared to the control. A higher GT dose, HGT 5X, and HGT 10X have significantly higher forelimb-grip strength than LGT 1X and MGT 2X. This suggests that the potential benefits of GT administration have a beneficial effect on increasing grip strength without training. Further, GT extract significantly improved the endurance-swimming time in a time-dependent manner compared to the control. The administration of phytochemicals, such as resveratrol, capsaicin, and curcumin, has been shown to improve the forelimb-grip strength and endurance-swimming time in animal models without training [22,28,29]. Supplementation of Chinese herbal extract from Cornu cervi pantotrichum increased the swimming time and forelimb-grip strength [30].

Apart from swimming tests, blood biochemical parameters are also used as a marker for fatigue. Glucose is produced from tissue glycogen in the liver and muscle and released in blood for the energy source [31]. High-intensive exercises have a high energy demand and consume glucose from tissue glycogen and increase their concentration in the blood [32]. Low-dose GT extract (LGT 1X) does not alter the serum glucose level significantly after the exercise. However, in MGT 2X, HGT 5X, and HGT 10X, the glucose levels were significantly increased after exercise when compared to the control. Thus, GT extract increases exercise performance by supplying/maintaining high glucose levels in the blood. Blood lactate is the metabolic end-product during glycolysis of carbohydrates under anaerobic conditions, and anaerobic glycolysis serves as a key energy source during intensive exercise [33]. Accumulated lactic acid during intensive exercise in the blood decreases the pH of muscles, and blood can cause acidosis. Acidosis is known to damage muscle and other vital organs and cause fatigue [34]. BUN is a metabolic product of protein metabolism. In amino acid metabolism, ammonia is produced as a metabolic end-product. BUN and ammonia accumulation increased during high-intensity exercise, reducing the performance and causing fatigue [35]. Preventing the accumulation and removal of lactic acid, BUN, and ammonia from the blood could increase exercise performance and prevent exercise-induce fatigue. In rats, *Moringa oleifera* extracts reduced the accumulation of LA, BUN, and ammonia and increased the endurance swimming time [36]. In our study, we found that GT extract does not alter BUN levels significantly in all groups. However, lactic acid and ammonia levels increased in LGT 1X, but higher GT doses, namely MGT 2X, HGT 5X, and HGT 10X, significantly decreased the lactic acid and ammonia levels. This suggests the potential anti-fatigue effect of GT.

Tissue glycogen from the muscle and liver is the key energy source during glycolysis and oxidative phosphorylation and increases the glucose level in blood during physical exercise [27]. High levels of hepatic and muscle glycogen could increase endurance time and performance during exercise with high intensity and reduce fatigue [37]. Compounds increasing the tissue glycogen level could have an anti-fatigue effect. *Antrodia camphorata*–extract treatment in mice increased the hepatic and muscular glycogen level and increased the endurance swimming time [38]. GT treatment in the mice increased the muscle and liver glycogen levels.

Liver function marker alanine aminotransferase (ALT); aspartate aminotransferase (AST); kidney function marker creatinine and uric acid; and other organ markers, such as creatine kinase (CK), creatine phosphokinase (CPK), and lactate dehydrogenase (LDH), are key markers for tissue damage during high-intensity exercise [39]. *Antrodia camphorata* extract, an edible fungus extract with high triterpenoids content has an anti-fatigue effect by increasing glucose level reducing the accumulation of BUN and lactic acid in blood and decreasing CK activity [38]. In our study, GT-extract treatment does not change the activity of these enzymes, suggesting no organ damage during exercise performance. The presence of phenolic compounds, such as luteolin and luteolin-7-glucoside, in the GT extract has been shown to have a hepatoprotective effect [14,18].

Fats are the second macronutrient that gives energy after glucose. During exercise, lipolysis is a major contributor to elevated triglyceride levels during rest [40]. Some natural phytochemicals induce fat/lipid metabolism, which delays the glycogen as an energy source. Aqueous extract of *Millettiae speciosae* Champ. supplementation increased fat utilization and reduced triglyceride and muscle glycogen usage as an energy source; thus, it possesses an anti-fatigue effect [41]. However, in our present study, GT extract did not change the lipid profile. Apart from forelimb-grip strength, endurance-swimming time and biochemical parameters related to exercise-induced fatigue, histological analysis of liver, kidney, heart, muscle, lung, and testis tissue were used to evaluate the impairment induced during exercise [42]. GT administration does not cause any morphological abnormalities in the liver, kidney, heart, muscle, lung, and pancreas.

## 5. Conclusions

*G. tenuifolia* is a traditional antipyretic and hepatoprotective herb used in Penghu Island and may soon become an important economic healthy food. However, the information on effective ingredients was still rare and unclear until now. In our previous study, some active ingredients, including luteolin and luteolin-7-glucoside, were reported in the GT extract [14,18]. Safety reports of the GT extract have also shown no toxicity of up to 10 g/b.wt. The experimental results show that this plant extract could be used for formulating food supplementation/health drinks exhibiting anti-fatigue effect and improving exercise performance.

In conclusion, GT extract treatment in mice could enhance physical performance, including forelimb-grip strength and endurance-swimming time. Furthermore, fatigue blood biochemical parameters indicate that GT extract improves glucose level and prevents the accumulation of lactic acid, BUN, and ammonia from the blood. GT possesses an anti-fatigue effect by increasing tissue glycogen levels in the liver and muscle. Taken together, herbal extract GT can be used to mitigate fatigue during exercise and increase exercise performance.

## Figures and Tables

**Table 1:** Bodyweight and organ weight of the treatment group.

Parameters	Control	LGT 1X	MGT 2X	HGT 5X	HGT 10X
Final BW (g)	40.92 ± 0.68	41.88 ± 0.63	41.86 ± 0.63	42.22 ± 0.39	39.87 ± 0.23
Muscle (g)	0.419 ± 0.010 ^b^	0.402 ± 0.013 ^b^	0.420 ± 0.016 ^b^	0.400 ± 0.009 ^ab^	0.368 ± 0.009 ^a^
Liver (g)	1.585 ± 0.050 ^ab^	1.558 ± 0.100 ^ab^	1.575 ± 0.056 ^ab^	1.845 ± 0.298 ^b^	1.358 ± 0.037 ^a^
Heart (g)	0.187 ± 0.006 ^a^	0.207 ± 0.012 ^ab^	0.219 ± 0.014 ^b^	0.208 ± 0.008 ^ab^	0.180 ± 0.008 ^a^
Pancreas (g)	0.226 ± 0.007 ^c^	0.189 ± 0.013 ^b^	0.186 ± 0.009 ^b^	0.152 ± 0.015 ^a^	0.175 ± 0.007 ^ab^
Lung (g)	0.220 ± 0.005 ^a^	0.211 ± 0.003 ^a^	0.212 ± 0.005 ^a^	0.208 ± 0.004 ^a^	0.216 ± 0.003 ^a^
Kidney (g)	0.580 ± 0.013 ^bc^	0.533 ± 0.016 ^ab^	0.592 ± 0.033 ^c^	0.527 ± 0.017 ^a^	0.484 ± 0.014 ^a^
Spleen (g)	0.1081 ± 0.0035 ^b^	0.1093 ± 0.0048 ^b^	0.0974 ± 0.0049 ^ab^	0.0898 ± 0.0050 ^a^	0.0902 ± 0.0029 ^a^
Testis (g)	0.262 ± 0.016 ^a^	0.269 ± 0.008 ^a^	0.245 ± 0.010 ^a^	0.237 ± 0.010 ^a^	0.246 ± 0.009 ^a^
Epididymis (g)	0.116 ± 0.020	0.087 ± 0.009	0.094 ± 0.015	0.068 ± 0.010	0.059 ± 0.002
Epididymis fat (g)	0.97 ± 0.1 ^b^	0.77 ± 0.0 ^a^	0.74 ± 0.1 ^a^	0.76 ± 0.0 ^a^	0.82 ± 0.1 ^ab^

Data are the mean ± SEM for *n* = 10 mice in each group. Values in the same row with different superscript letters (^a^, ^b^, and ^c^) differ significantly, *p* < 0.05.

**Table 2:** Biochemical analysis at the end of the treatment period in treatment groups.

Parameters	Control	LGT 1X	MGT 2X	HGT 5X	HGT 10X
GOT (U/L)	117.9 ± 18.8 ^a^	101.5 ± 6.8 ^a^	116.2 ± 15.1 ^a^	140.8 ± 24.5 ^ab^	176.3 ± 29.5 ^b^
GST (U/L)	27.8 ± 2.8 ^a^	23.0 ± 1.0 ^a^	26.6 ± 3.6 ^a^	44.9 ± 16.6 ^a^	28.0 ± 2.1 ^a^
Triglyceride (mg/dL)	66.8 ± 6.1 ^a^	90.1 ± 15.5 ^a^	84.8 ± 7.9 ^a^	75.2 ± 6.8 ^a^	80.1 ± 7.2 ^a^
Total cholesterol (mg/dL)	153.7 ± 6.4 ^a^	154.6 ± 5.9 ^a^	142.7 ± 6.5 ^a^	151.6 ± 5.4 ^a^	153.7 ± 8.0 ^a^
HDL (mg/dL)	117.7 ± 4.29 ^a^	120.8 ± 5.63 ^a^	114.0 ± 5.58 ^a^	122.5 ± 4.09 ^a^	123.9 ± 7.24 ^a^
LDL (mg/dL)	25.0 ± 2.09 ^b^	22.1 ± 1.72 ^ab^	19.8 ± 1.28 ^a^	20.9 ± 0.98 ^ab^	19.5 ± 1.42 ^a^
BUN (mg/dL)	24.9 ± 0.70 ^a^	27.3 ± 1.32 ^ab^	28.6 ± 1.74 ^bc^	31.0 ± 0.77 ^c^	25.9 ± 1.55 ^ab^
Creatinine (mg/dL)	0.228 ± 0.014 ^a^	0.225 ± 0.017 ^a^	0.235 ± 0.022 ^ab^	0.214 ± 0.014 ^a^	0.277 ± 0.014 ^b^
Uric acid (mg/dL)	2.44 ± 0.20 ^ab^	3.01 ± 0.22 ^b^	2.13 ± 0.18 ^a^	2.00 ± 0.24 ^a^	2.35 ± 0.24 ^a^
Glucose (mg/dL)	131.7 ± 10.8 ^a^	160.5 ± 5.3 ^bc^	152.3 ± 6.7 ^ab^	166.9 ± 11.1 ^c^	138.9 ± 6.6 ^ab^
CPK (U/L)	5789 ± 1495 ^ab^	3420 ± 490 ^a^	4762 ± 921 ^a^	6019 ± 1632 ^ab^	9168 ± 1841 ^b^
LDH (U/L)	1214 ± 193 ^a^	721 ± 134 ^a^	810 ± 190 ^a^	924 ± 259 ^a^	1012 ± 111 ^a^
Ammonia (μM/L)	414 ± 87 ^a^	485 ± 106 ^a^	632 ± 132 ^a^	528 ± 124 ^a^	645 ± 103 ^a^

Data are the mean ± SEM for *n* = 10 mice in each group. Values in the same row with different superscript letters (^a^, ^b^, and ^c^) differ significantly, *p* < 0.05.

## Data Availability

The data that support the findings of this study are available upon request from the corresponding author.
